# The Role of Autophagy in Hepatocellular Carcinoma

**DOI:** 10.3390/ijms161125984

**Published:** 2015-11-06

**Authors:** Yoo Jin Lee, Byoung Kuk Jang

**Affiliations:** Division of Gastroenterology and Hepatology, Department of Internal Medicine, Keimyung University School of Medicine, Daegu 700-712, Korea; doctorlyj@naver.com

**Keywords:** hepatocellular carcinoma, autophagy, tumorigenesis, tumor suppression, prognosis, therapy

## Abstract

Autophagy is a catabolic process involved in cellular homeostasis under basal and stressed conditions. Autophagy is crucial for normal liver physiology and the pathogenesis of liver diseases. During the last decade, the function of autophagy in hepatocellular carcinoma (HCC) has been evaluated extensively. Currently, autophagy is thought to play a dual role in HCC, *i.e.*, autophagy is involved in tumorigenesis and tumor suppression. Recent investigations of autophagy have suggested that autophagy biomarkers can facilitate HCC prognosis and the establishment of therapeutic approaches. In this review, we briefly summarize the current understanding of autophagy and discuss recent evidence for its role in HCC.

## 1. Introduction

Autophagy is a catabolic process with crucial roles in development, differentiation, homeostasis, and the survival of cells in nutrient-deprived conditions [1,2]. There have been three major modes of autophagy: macroautophagy, microautophagy, and chaperone-mediated autophagy [3,4]. Macroautophagy is usually referred to as “autophagy” owing to the limited data for the other forms [4]. Upon induction, the isolated membrane or phagophore wraps around portions of the cytoplasm to form a double-membraned vesicle known as an autophagosome [1]. Autophagosomes which subsequently fuse with lysosomes are degraded by lysosomal proteases during maturation [5]. In addition to preserve intracellular metabolic homeostasis, autophagy is induced in response to starvation, protein aggregation, and other forms of stress such as oxidative and endoplasmic reticulum (ER) stress [6,7].

Abundant evidence has revealed that autophagy is involved in the pathogenesis of various diseases, such as neurodegenerative diseases, infectious diseases, metabolic diseases, and cancers [4,8,9,10,11]. Increasing studies have indicated the crucial role of autophagy in liver diseases. The dysregulation of autophagy is associated with viral hepatitis, non-alcoholic fatty liver disease, alcoholic liver disease, fibrosis, cirrhosis, and hepatocellular carcinoma (HCC) [1,12]. Recently, many investigators have suggested that tumor cells rely on autophagy for survival in HCC, although it is still controversial whether autophagy serves as an anti-cancer or pro-cancer mechanism [13,14].

Although there are several treatment options for HCC, the effectiveness of most curative treatments is limited to the early stage of HCC [15]. Currently, there is no effective treatment for patients showing advanced- or intermediate-stage HCC [16]. Furthermore, dysregulation of apoptosis has been observed, which is associated with resistance to HCC treatment [17,18]. Thus, autophagy-related markers, including microtubule-associated protein 1 light chain 3 (LC3) and beclin-1, are potential prognostic factors for HCC [19,20]. In recent studies, autophagy-based therapies, such as hydroxychloroquine (HCQ) or chloroquine (CQ), have also been examined in mice xenograft models [21,22]. These autophagy-modulating compounds have potential applications in the treatment of HCC in the near future. In this review, we provide a brief overview of autophagy and HCC, with focusing on: (1) the current understanding of autophagy pathways; (2) autophagy function in HCC; and (3) prognostic and therapeutic clinical applications in HCC.

## 2. Autophagy

Autophagy is a highly conserved process consisting of sequential stages: initiation, elongation, autophagosome formation, and autophagosome fusion with lysosomes then degradation [23]. These steps are regulated by autophagy-related genes (Atgs). To date, more than 30 Atgs have been identified and their functions have been evaluated extensively. In particular, experiments using specific Atg-deletion models in the liver provide evidence of the critical roles of autophagy in adaptive responses to starvation and various forms of stress, homeostasis, and cellular differentiation and development [24,25,26,27,28]. The details of the autophagy process are presented below. (i) The initiation stage is regulated by the adenosine monophosphate-activated protein kinase (AMPK), UNC51-like kinase 1 (ULK1) and mammalian target of rapamycin complex 1 (mTORC1) complexes. mTORC1 is the main inhibitor of autophagosome formation by ULK1. Under nutrient starvation such as glucose, the activated AMPK inhibits mTORC1, then directly phosphorylates ULK1, and leads to autophagy initiation [29,30]; (ii) Nucleation of the phagophore is mediated by the Beclin-1-class III phosphatidylinositol 3-kinase (PI3K) complex that includes Beclin-1, Vps34 (class II PI3K), p150 (homolog of Vps15), Atg14L/Barkor, and Ambra-1 [4]; (iii) Elongation of the phagophore into a complete autophagosome is regulated by two ubiquitin-like protein conjugated complexes: Atg5-Atg12-Atg16L1 and LC3-II. Several Atgs, such as E1-like protein, Atg7, E2-like protein, and Atg 10, are necessary mediators of these processes [31,32,33]. LC3 is the major mammalian ortholog of Atg8. LC3-1 is converted to LC3-II and degraded after autophagosomes fuse with lysosomes [34]. Thus, LC3-II is considered an autophagosome marker [10]. Microautophagy can engulf cargo nonselectively (through random sequenstation) or selectively (by individual targeting of each cargo molecule) [35]; (iv) The final stage is autophagic degradation. As there are many excellent reviews regarding the autophagy process, reading those reviews will be helpful for further understanding of the autophagic pathway [36,37].

## 3. The Role of Autophagy in the Liver

Several selective modes of autophagy, mitophagy [38,39] and lipophagy [25], were first suggested based on experiments with cultured hepatocytes and whole livers. Since then, considerable data related to the role of autophagy in the liver has accumulated. Autophagy is involved in diverse liver physiology and pathophysiology, e.g., clearing misfolded proteins, nutrient and energy metabolism in hepatocytes, regulating selective organelle degradation, lipid and alcohol metabolism, and hepatitis virus infection [40].

The liver highly relies on the autophagy for its physiology and pathology. In particular, lysosome-mediated degradation is crucial in both normal physiological conditions and in stress responses, such as in proteotoxicity, metabolic dysregulation, infection, and carcinogenesis [35]. Therefore, the dysfunction of autophagy is associated with various liver diseases, suggesting that the regulation of this process is a potential therapeutic approach [40].

Viral infections, such as chronic hepatitis B or C infections, and alcohol abuse are frequent etiologic factors of HCC [16]. In these conditions, multiple steps are involved in the development of HCC: liver cell death, inflammation, liver cell proliferation, liver fibrosis, and finally HCC development [41]. Non-alcoholic fatty liver disease, diabetes, ingestion of aflatoxin B1, chronic alcohol abuse, obesity, and genetic disorders also can be risk factors of HCC [42]. Autophagy is deeply involved in both the above mentioned etiologic factors and HCC itself. In mice models of obesity, the suppressed hepatic autophagy such as Atg is causal to impaired hepatic sensitivity and glucose homeostasis [43]. The details regarding the mechanisms of autophagy in the liver are well reviewed elsewhere [1,37,40,44].

## 4. Dual Role of Autophagy in Hepatocellular Carcinoma

**Figure 1:**
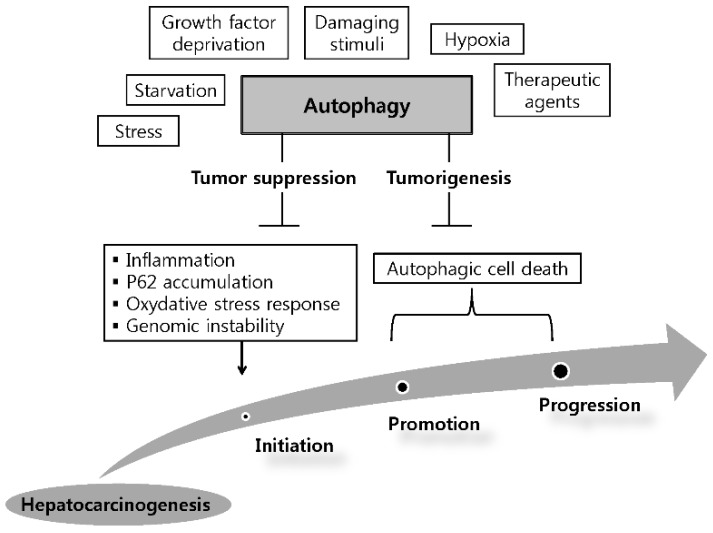
Dual role of autophagy in the initiation and development of hepatocellular carcinoma. Hepatic autophagy is activated by various factors. By limiting inflammation, P62 accumulation, oxidative stress response and consequently inhibiting genomic instability, autophagy can serve as a tumor suppressor in the initiation stage of hepatoneogenesis. On the other hand, autophagy can facilitates tumorigenesis via autophagic cell death in other stages of hepatoneogenesis.

Although the mechanism of autophagy has been extensively investigated, the actual functions of autophagy in HCC are still largely unknown. The dual role of autophagy in the development and promotion of HCC has been suggested as a model for the autophagy in HCC, *i.e.*, it is involved in tumorigenesis and tumor suppression (Figure 1).

Early studies have demonstrated a tumor suppressive function of autophagy, while more recent studies have revealed a tumorigenesis function [35]. In normal cells, autophagy inhibits tumorigenesis by removing damaged organelles and aggregated proteins. By contrast, in tumor cells, autophagy serves the survival of tumor cells via the following mechanisms: (1) promotion of metabolite turnover and absorption in tumor cells; (2) inhibition of apoptosis and reactive oxygen species production; and (3) increasing drug resistance [45]. Abundant evidence supports these two functions of autophagy in HCC carcinogenesis. Understanding the involvement of autophagy in HCC is crucial because it may facilitate the development of future therapeutic approaches to HCC.

### 4.1. Tumor Suppressive Function of Autophagy

One of the earliest and representative results supporting the tumor suppressive role of autophagy was related to Beclin-1-knockout mice. Mice with homozygous knockout of Becliln-1 have markedly reduced autophagic activity and a highly prevalent cancer such as HCC [46]. Similarly, other study has shown that the mice model of Beclin-1 heterozygous disruption leads to increased frequency of malignancies and promotes the development of premalignant lesions. Additionally, heterozygous disruption of Beclin-1 leads to increased cellular proliferation and reduced autophagy [47].

Particularly convincing evidence for the tumor-suppressive role of autophagy is the development of multiple liver adenomas in mice with a deletion of Atg5 and Atg7 [28]. Notably, Atg5 mosaic knockouts develop tumors only in the liver, but not in other tissues, suggesting that hepatocytes have a dependence on the tumor-suppressive role of autophagy [28]. Atg5 and Atg7 constitute two ubiquitin-like conjugation systems, which are required for the formation of autophagosome [26]. It is noteworthy that Beclin-1- and Atg5/7-knockout mice have different phenotypes. For example, Atg5- and Atg7-knockout mice survive until birth [26,48]; however, Beclin-1-knockout mice die during early embryonic development [46]. These data indicate that Beclin-1 might have other complex functions in addition to autophagy.

The stress-inducible intracellular protein p62 targets the autophagosome formation site on ER [49]. It directly interacts with LC3, and is merged to the autophagosome and degraded by autophagy [50]. It is involved in multiple cellular functions, including tumorigenesis [51]. Some studies have shown that accumulated p62 via an autophagy deficiency results in tumor development and progression. Autophagy-defective tumor cells accumulate p62 in response to stress, contributing to hepatocarcinogenesis via NF-kB regulation and gene expression. Suppressing p62 accumulation prevents defective autophagy-related damage, suggesting that a failure to regulate p62 causes oxidative stress [52]. In mice with liver-specific knockouts of Atg7, the growth of liver adenomas is strongly suppressed in combination with p62 knockouts [28]. Together, these results establish a crucial role for autophagy as a tumor suppressor in HCC.

### 4.2. Autophagy as a Tumorigenesis Mechanism

To sustain their rapid proliferation, cancer cells need abundant nutrients and oxygen during progression and invasion. As a key regulator for cellular homeostasis, autophagy is related to the maintenance and survival of tumor cells [53]. Since autophagy is activated in tumor cells in response to various types of stress, it plays a pro-survival function of cancer cells [11,54]. Cancer cells are thought to increase autophagic activity to survive in hostile microenvironments [55].

For example, mRNA of LC3 is markedly higher in HCC tissues than in non-tumor parenchymal cells, and is significantly correlated with tumor size [56]. Additionally, LC3-II increased in human HCC tissues showing decreased glucose uptake and increased K-Ras expression [57]. The tumorigenesis mechanism is also partly supported by a study that growth factor-deprived animal cells maintained cell survival via autophagy [58]. Furthermore, increased autophagic activity has been observed in hypoxic tumor regions [59].

The tumorigenesis function of autophagy is a promising target of future research focused on cancer management. Several agents that block the autophagy process, such as CQ and HCQ, have been actively studied in pre-clinical studies and clinical trials. The details of these studies are presented in the subsequent section.

## 5. Autophagy as a Prognostic Factor for HCC

HCC is one of the most aggressive and lethal tumors worldwide. It shows a very low 5-year survival rate (<15%) owing to its late diagnosis and compromised underlying liver function [60]. Patients who are eligible for current curative therapies are limited, and alternative therapies do not improve patient survival [15]. Thus, prognostic factors and promising therapies for HCC need to be established. Investigations have identified that the dysregulation of autophagy is associated with tumorigenesis in diverse types of cancer [10,61,62,63]. The role of autophagy in HCC is relatively well established [14]. However, few studies have identified autophagic proteins that are prognostic parameters in HCC, and these should be further elucidated considering the crucial role of autophagy during carcinogenesis. Here, we summarize the existing autophagy-related markers, which have prognostic potential in HCC. Autophagy-related markers that are predictive for the prognosis of HCC are presented in Table 1.

**Table 1:** Autophagy-related markers predictive for prognosis of hepatocellular carcinoma.

Markers	Target Mechanism	Expression Level in Human HCC Tissue	Expression Assessed	Implication	References
Beclin-1	Autophagosome formation	↓	protein	↑: Good prognosis	[20]
		↓	protein	↓: Aggressive behavior	[64]
		↓	mRNA, protein	↑: Good prognosis	[65]
		↓	mRNA, protein	↓: Progression of tumor	[66]
Microtubule-associated protein 1 light chain 3 (LC3)	Autophagosome formation	↓	protein	↑: Good prognosis	[19]
		↑	protein	↑: Poor prognosis	[67]
		↑	protein	↑: Poor prognosis	[68]
		↑	protein	↑: Poor prognosis	[69]
		↓	protein	↑: Poor prognosis	[70]
		↓	protein	↑: Good prognosis	[71]
Stearoyl-CoA desaturases (SCD1)	Adenosine monophosphate-activated protein kinase (AMPK) signaling	↑	mRNA, protein	↑: Poor prognosis	[71]
CCAAT-enhancer-binding protein α (C/EBPa)	Transmembrane protein 166 (TMEM166)	↑	protein	↑: Poor prognosis	[72]
GABA receptor-associated protein like 1 (GABARAPL1)	Autophagosome formation	↓	mRNA, protein	↓: Poor prognosis	[73]
UNC51-like kinase 1 (ULK1)	Autophagosome formation	↑	protein	↑: Poor prognosis	[74]
Gankyrin	Upregulate Atg7	↑	mRNA, protein	↑: Poor prognosis	[75]

↑, increase; ↓, decrease.

### 5.1. Beclin-1

Beclin-1, the mammalian ortholog of yeast Atg6, is required for double-membrane autophagosome formation [76,77]. It promotes the initial stages of autophagy via binding with other autophagic proteins, including Bcl-2, p150, Vps34, Atg14L, ultraviolet irradiation resistance-associated gene (UVRAG), Rubicon, and Bif1, to form a huge protein complex [78,79,80,81,82]. In fact, the identifying Beclin-1 as a tumor suppressor gene in human cancer provided early evidence for the tumor suppressive role of autophagy [83]. In a microarray analysis of HCC tissues obtained at the time of hepatectomy, investigators found lower expression of Beclin-1 in HCC than in adjacent nontumorous tissue, indicating that defective autophagy can contribute to carcinogenesis [64,65]. Studies have demonstrated that aberrant expression of Beclin-1 in diverse kinds of cancer is linked with poor prognosis [84,85,86]. Consistent with these findings, increased Beclin-1 expression is correlated with better prognosis, such as longer disease-free survival and overall survival (OS), in HCC [65]. The association between higher expression of Beclin-1 and longer OS supports the use of Beclin-1 as a prognostic marker for HCC. Additionally, Beclin-1 was proposed as a marker of HCC progression as co-expression of related genes was linked to HCC tumor progression [20]. A more recent study has confirmed the role of Beclin-1 in the progression of HCC by revealing a significant association between reduced Beclin-1 and high HIF-1α expression [64]. Combined, Beclin-1 is expected to be a valuable prognostic marker of HCC.

### 5.2. LC3

LC3, the mammalian homolog of yeast Atg8, plays an important role in human autophagy. It is activated by an ubiquitination-like reaction that is regulated by Atg7 and Atg3 [87]. The LC3 proform is cleaved into soluble LC3-I and lipidated LC3-II. LC3-I is converted to LC3-II, which is recruited for autophagosome formation [32]. Thus, LC3-II is an indicator of autophagosome formation [34]. However, LC3-II can reflect either autophagy induction or suppression [2]. The link between aberrant expression of LC3 and cancer prognosis has been reported for several tumor types [88,89,90,91]. A recent study suggested that high expression of a stone-like pattern of LC3A is an independent predictor of HCC prognosis [68]. Similarly, we identified LC3 expression as an independent prognostic factor of OS and time to recurrence. Interestingly, LC3 expression at an advanced stage is correlated with a longer OS, but this correlation is not observed in the early stage of HCC. These results imply that LC3 plays different roles depending on tumor stage [19]. The prognostic role of LC3 in HCC has been supported by several studies [67,69,70]. Collectively, these results indicate that LC3 is a potential prognostic factor in patients with HCC; however, its application as a potential therapeutic target for HCC requires additional research.

### 5.3. ULK1

ULK1, a mammalian serine/threonine protein kinase, contributes to the initial stages of autophagy [74]. There are conflicting data regarding the association between ULK1 and cancer prognosis. For instance, increased expression of ULK1 is associated with poor prognosis in esophageal squamous cell carcinoma [92]; however, in breast cancer, decreased ULK1 expression is associated with poor prognosis [93]. For HCC, one study showed that increased ULK1 expression is associated with poor OS, indicating that it is a possible prognostic marker for HCC [74]. Taken together, autophagy acts differently for different cancer types.

### 5.4. GABARAPL1

GABA receptor-associated protein like 1 (GABARAPL1) is one of six human Atg8 family proteins that shares high sequence identity with GABARAP, GATE-pro15, and LC3 [94]. Studies have revealed that GABARAPL1 is involved in autophagosome formation [94,95]. Some evidence suggests that GABARAPL1 expression is lower in HCC tissues than in adjacent non-tumor tissues and that there is an association between low GABARAPL1 expression and poor prognosis in HCC patients [73]. GABARAPL1 has previously been associated with the risk of metastasis in breast adenocarcinoma [96]. Further studies are required to understand the detailed mechanism of GABARAPL1 in HCC carcinogenesis and progression.

### 5.5. Other Loci Associated with Autophagy

Several additional autophagy-associated markers have been proposed for HCC prognosis. Stearoyl-CoA desaturases (SCDs) are ER-bound enzymes involved in *de novo* fatty acid synthesis of cellular lipids [97]. It regulates autophagy via lipogenesis [98]. Huang *et al*. [71], showed that SCD1 expression is significantly increased in mRNA and protein of HCC tissues and both LC3 and SCD1 were identified as independent predictors of OS in HCC. They also suggested that the tumor suppressive roles of SCD1 negatively modulate autophagy-induced apoptosis through the activation of AMPK signaling in human HCC.

CCAAT-enhancer-binding protein α (C/EBPα) is a transcription factor which belongs to the CCAAT/enhancer-binding protein family that inhibits cell proliferation and promotes terminal differentiation in various cell types [99]. It is involved in glucose and lipid metabolism [100]. Lu *et al.* [101] reported that C/EBPα is upregulated in protein of human HCC and promotes cell growth in HCC cell lines. They also demonstrated that C/EBPα overexpression is an independent prognosticator of poor OS in HCC patients [72]. It is noteworthy that C/EBPα is related to autophagy-mediated lipid metabolism and resistance to energy starvation, which lead to HCC carcinogenesis [72]. These results provide important molecular evidence for future therapeutic targets of HCC.

Gankyrin (also known as PSMD10 or p28) is a small protein that was identified as a regulatory subunit of the 26S proteasome complex [102,103]. Although the detailed mechanism is poorly understood, Gankyrin is overexpressed in mRNA and protein of HCC and acts as an oncoprotein [103,104]. A study reported that Gankyrin promotes autophagy in response to starvation or stress in HCC. Moreover, the significant correlation between Gankyrin and Atg7 indicates that the combination of these two molecules might be used in the prognosis of HCC [75]. The precise impact of Gankyrin on the prognosis of patients with HCC requires further investigation.

## 6. Autophagy Modulation for HCC Therapy

As autophagy acts a dual role in the initiation and development of HCC, many researches have evaluated its mechanisms and applications to HCC treatment. Increasing evidence supports the fact that autophagy also contributes to tumor cell responses to therapies and changing environmental stimuli. In this section, we discuss important autophagy inhibitors and inducers in HCC.

### 6.1. Autophagy Inhibitors in Anti-HCC Therapy

Since autophagy maintains cellular homeostasis, cancer cells also use autophagy for survival against cellular stress, including anti-HCC therapies. Autophagy inhibition prohibits the pro-survival effect of autophagy and enhances cytotoxicity in combination with anti-HCC therapies [105]. CQ and HCQ, which are used in the treatment of malaria, inhibit autophagy by increasing pH of lysosome via sinking protons. Eventually, CQ and HCQ are able to block the final stage of autophagy that demands acidic lysosomes for degradation [106]. It has been reported that autophagy inhibitors can potentiate the efficacy of oxaliplatin, cisplatin, 5-FU, and sorafenib in HCC [22,107,108,109]. The combination of oxaliplatin with CQ induces marked tumor suppression compared with either agent alone in HCC xenografts [22]. Similarly, the co-administration of CQ and sorafenib leads to marked tumor suppression in an HCC cell line [109]. MicroRNAs, which are small non-coding RNAs, have been proposed as key players in cancer cell proliferation, tumorigenesis, and apoptosis in HCC [110,111]. A recent study showed that miR-375 is downregulated in HCC tissues and inhibits hypoxia-induced autophagosome formation and autophagic flux in HCC cells [112]. Additionally, small interfering RNAs inhibit specific autophagy functions by silencing Atgs, and promote chemotherapeutic agent-induced cell death in a HCC cell line [113,114]. Zoe *et al*. [115], reported that oroxylin A which is a natural mono-flavonoid extracted from *Scutellariae radix*, leads to Beclin-1-mediated autophagy in human HCC cells. An *in vivo* study demonstrated that oroxylin A impedes the growth of xenograft tumors and causes apparent autophagy in tumors. The clinical application of these autophagy inhibitors remains unclear, but they are a promising therapeutic strategy to overcome therapeutic resistance in HCC treatment. Although there are over thirty ongoing clinical trials targeting autophagy for cancer treatment, none focus on HCC. Accordingly, clinical trials targeting autophagy for HCC therapy are required.

### 6.2. Autophagy Inducers in Anti-HCC Therapy

Based on the protective function of autophagy against hepatocarginogenesis, many researchers have focused on tumor cell death by autophagy. Autophagic cell death has been identified in many cancer cells [116,117,118]. The PI3K/Akt/mTOR pathway is a main regulator of cell proliferation, growth, survival, protein synthesis, and glucose metabolism in cancer cells [119]. In this pathway, mTOR inhibitors show anti-tumor activity in HCC [120]. The mTOR pathway is upregulated in a significant number of HCCs, suggesting that targeting this pathway may be particularly beneficial in the treatment of HCC [121]. Rapamycin and its derivatives act as mTOR inhibitors and are common autophagy inducers. For instance, rapamycin showed an anti-tumoral effect in a phase II study with 25 patients with advanced HCC [122]. Other study showed that rapamycin-based immunosuppression is relate to improved patient OS after liver transplantation for HCC [123]. However, it is premature to generalize about the utility of rapamycin and its derivatives in HCC therapy owing to insufficient or conflicting data for each agent. For example, everolimus (RAD001) shows anti-tumor activity in xenograft models of human HCC [124]; however, a recent large study (EVOLVE-1 trial) revealed that it had no benefit for survival and disease progression in a phase III trial [125]. By contrast, more fully targeting mTOR with RAD001 and a PI3K/mTOR dual inhibitor showed greater efficacy by increasing autophagy/mitophagy in tumors and decreasing tumor size in a mouse model of HCC [126]. Interestingly, a more recent study suggested that compound SBI-0206965 which is a highly selective ULK1 inhibitor may have promise as a more specific inhibitor of autophagy in the treatment of HCC, potentially in combination with mTOR inhibitor [127]. Further studies supporting clinical implication of rapamycin are necessary.

Sorafenib is a multi-kinase inhibitor that is used a first-line systemic therapy for advanced HCC. It promotes autophagic cell death via the Mcl-1 signaling pathway [122]. Currently, representative phase III trials have shown that sorafenib significantly improves overall survival in patients with advanced HCC [128,129]. The co-administration of sorafenib and autophagy inhibitors also shows improved efficacy via autophagic cell death. However, sorafenib-induced autophagic cell death can result in drug resistance in patients with HCC [130]. Further, conventional chemotherapeutics, such as doxorubicin, are suppressed by sorafenib, resulting in cell progression, increased survival, and decreased autophagy in tumor cells [131]. Future research regarding the application of autophagy inducers to ameliorate the current limits of HCC therapy should facilitate better treatment outcomes in HCC.

## 7. Conclusions

The liver plays a crucial role in metabolism; therefore, dysregulation of liver autophagy has great clinical implications. Extensive studies have revealed the involvement of autophagy in HCC. According to current knowledge, autophagy plays a dual role in HCC. Most studies support the tumor suppressive role of autophagy. However, recently, abundant evidence suggests that autophagy is involved in tumorigenesis. We presume that the heterogeneousity of HCC tumors partly contributes to the different role of autophagy in various populations of HCC cells. Thus, the complexity of autophagy in HCC requires individualized approaches for the management of HCC.

Although abundant evidence suggests the high potential for autophagy modulation as a therapeutic method for HCC, the clinical application of these autophagy modulators remains unclear. Much has yet to be determined about the diverse and complex process of autophagy. A promising challenge is to identify the specific molecular mechanisms of autophagy for the development of therapeutic targets and to overcome resistance to current therapies. Another challenge is to identify biomarkers that can differentiate patients who need early intensive treatment and meticulous surveillance. Additional research on the various functions of autophagy according to tumor stage, differentiation, and environmental and genetic factors may lead to the establishment of an effective strategy against HCC.
